# Concurrent analysis of histomorphology and immune-related gene expression in gills of Atlantic salmon (*Salmo salar*) from recirculating aquaculture systems

**DOI:** 10.1016/j.bbrep.2026.102808

**Published:** 2026-10-03

**Authors:** Thomas Amlie, Simen Foyn Nørstebø, Rakesh Das, Cheng Xu, Øystein Evensen

**Affiliations:** a DNV Aquaculture and Ocean Health, Sistranda, Norway; b Norwegian Veterinary Institute, PO Box N-5000, Ås, Norway; c Norwegian University of Life Sciences, Faculty of Veterinary Medicine, PO Box 5003, Ås, N-1432, Norway

**Keywords:** Gill changes, Salmon, Histology, Gene expression, RAS

## Abstract

Gill health is crucial in aquaculture but remains difficult to define because the gills are dynamic organs with nonspecific responses to environmental and biological stressors. This descriptive, exploratory study examined histological changes and gene expression patterns in selected apparently healthy Atlantic salmon from two commercial recirculating aquaculture systems (RAS), encompassing freshwater and saltwater production phases. The two systems also differed in facility, fish size, and life stage. Using a semiquantitative histology scoring system and RT-qPCR, we assessed associations between mild gill alterations, including increased mucous cell numbers, lamellar clubbing, and inflammation, and the expression of selected immunological and physiological markers. The selected material included fish with and without apparent morphological changes, allowing exploratory examination of gene-morphology relationships across the included spectrum, but not estimation of the population prevalence of gill alterations. Within this material, mucous cell, lamellar clubbing, and lamellar inflammation scores were higher among fish from the saltwater production system. Associations that remained significant after correction for multiple testing included lamellar clubbing with IFN-α and IgT, lamellar inflammation with IgT, mucous cell numbers with IgM, and mucous cell numbers with NKA α-1a. Ca. *Branchiomonas cysticola* was the only pathogen detected and coincided with increased lamellar inflammation at subclinical loads. These exploratory findings characterize morphological and transcriptional variation within the selected sample and demonstrate the value of combining histology, gene expression, and pathogen detection when assessing gill health in RAS-reared salmon.

## Highlights

• Gill histomorphology and immune gene expression in apparently healthy RAS Atlantic salmon.
• Mild gill changes can be present, even in the absence of overt disease.
• Gene expression differed between the two production systems and fish groups.
• IFN-α and IgT track lamellar clubbing; IgT also tracks lamellar inflammation. IgM and NKA α-1a track mucous cell numbers.
• Ca. B. cysticola loads coincide with lamellar inflammation, but not causally.

## 1 Introduction

Gill health is fundamental to overall fish well-being. Yet, the definition of a healthy gill remains ambiguous and varies depending on the metrics used, biological function, structural integrity, fish behavior, or productivity [[1], [2], [3]]. Moreover, deviations in gill structure from what is considered “normal” do not necessarily imply compromised health [1].

A better understanding of typical variation in gill morphology in apparently healthy fish could significantly aid in determining the relevance of observed gill alterations to overall health. Such knowledge could also elucidate the progression from normal variability to clinical disease stages. In aquaculture, detecting negative trends in gill health during the subclinical phase is crucial, as it enables timely interventions that can prevent mortality under intensive farming conditions [4,5].

Gill disorders may have an infectious or non-infectious origin [6,7], although they are often described as multifactorial [[8], [9], [10], [11], [12]]. Various methods have been employed to evaluate different facets of gill health [1]. These include macroscopic scoring systems that categorize both non-specific and disease-specific alterations [[13], [14], [15]] and microscopic histological scoring systems that assess morphological changes at the histology level [3,9,13,14,16,17]. Molecular approaches are also utilized to detect pathogens and correlate gene expression with histopathological findings [13,14,16,18,19].

Molecular-level changes influence gill function and may precede morphological changes [20]. Numerous studies have explored the effects of external factors, including environmental conditions, biocides, anesthetics, and handling, on gill gene expression, as well as responses to infectious agents, including salmon gill pox virus [21,22].

Evaluating whether variation in gill gene expression in apparently healthy fish is reflected at the morphological level may provide insight into early or mild tissue responses. Characterizing co-occurring morphological and molecular patterns can help identify changes that merit further investigation. Representative sampling is, however, required to estimate the prevalence of such changes or to establish population-level reference ranges.

This study aimed to characterize gill morphology and the expression of selected immunological and physiological markers in apparently healthy Atlantic salmon from two commercial RAS production systems spanning freshwater and saltwater production phases. The systems differed not only in water type, but also in facility, fish size, life stage, and sampling time; comparisons between them are therefore descriptive rather than controlled estimates of environmental effects. We also assessed the presence of selected opportunistic pathogens using quantitative PCR (qPCR) and included fish classified as deviant on external or behavioral criteria for comparison. The study was designed to describe the range of morphology and gene expression represented in the selected material and to explore gene-morphology associations; it was not designed to estimate prevalence or define population-level “normal” baselines.

## 2 Materials and methods

### 2.1 Sample pool characteristics and target samples

Gill samples were collected from Atlantic salmon raised in two commercial RAS facilities with no suspicion of gill disease. Facility 1 reared fish in freshwater up to smolt size (approximately 150 g), whereas Facility 2 reared fish from smolt to market size in saltwater. Samples were collected over time from four fish groups, producing an apparently healthy sample pool comprising fish of different sizes, life stages, and RAS exposures; this pool has also been used in previous studies [23,24]. Throughout the manuscript, when we refer to ‘environments’ and comparisons between the two production systems, this encompasses differences in water environment, facility, fish size, life stage, and sampling time.

Apparently healthy fish were randomly netted and euthanized with an overdose of benzocaine. Fish with wound score 3 [25], pronounced emaciation (runts), or deviant behavior at sampling were excluded from the apparently healthy pool and classified as deviant. The smallest fish were euthanized as a group, whereas larger fish were occasionally crowded before netting for operational reasons. Immediately after euthanasia, the second gill arch on the left side was resected and, depending on fish size, either the entire arch or its ventral part was transferred to 10% formalin. Gill tissue for qPCR was collected from the second arch on the right side, or immediately dorsal to the left gill-arch angle in larger fish, and transferred to RNAlater. Samples for PCR were stored at −18°C or below. Formalin-fixed samples were routinely processed, sectioned at 2–4 μm, stained with H&E, and scanned to a digital image format by the same laboratory.

During the study period, 48 deviant fish from Facility 2 were sampled on an ad hoc basis between the routine sampling time points. In addition, six fish from the routine sampling were reclassified as deviant according to the criteria described above, yielding a total deviant sample pool of 54 fish. Of these, 42 were represented in the linked histology–RT-qPCR dataset used for the analyses. The sampling procedure was identical to that used for apparently healthy fish.

Four sampling time points were selected from the apparently healthy pool in each facility to represent notable differences in average fish weight (Table 1). At each selected time point, available histological sections were first screened at low magnification (2–5×) for technical suitability for the semiquantitative scoring method [23]. During this preliminary screen, sections were also noted as showing or not showing apparent morphological gill changes. The screen did not involve formal scoring, localization, or assessment of the type or severity of the changes.

**Table 1:** Sampling overview. The average fish weight is calculated per sampling time point based on the average weight at the tank level in Facility 1 and the individual fish weight in Facility 2. Deviant fish were not weighed during ad hoc sampling from December 2019 to June 2021.

Sample time point	Facility	Fish group (FG)	Average fish weight (g)	Sampled fish	The number of fish included in this study/for PCR analysis (n = 140)	Histological assessment^a^ (n = 127)
**March 2020**	1	1	11	20	12	12
**September 2020**		2	23	20	12	11
**November 2020**			69	20		9
**December 2020**			94	20		12
**January 2020**	2	3	769	21	12	11
**May 2020**			2297	20		10
**August 2020**		4	1291	10	10	10
**June 2021**			4010			7
**December 2019 – June 2021**	2	3 & 4	-	48	48	45

a The number of scored fish may differ from the selected number due to histology samples unsuitable for scoring and/or reclassifying 6 fish as deviant before analysis.

Samples previously included in the study by Amlie et al. [23] were retained automatically. To ensure sufficient morphological variation for exploratory gene-morphology analyses, we included up to three samples with and three without apparent changes at each time point, where possible. After these inclusions, the remaining samples required to reach a target of 10–12 fish were selected randomly in Microsoft Excel from the available material. For fish group 4, all fish from the selected time points were included. At some time points, histological sections considered less suitable during the preliminary screen were included to reach the target sample number. All fish from the deviant pool were included. Before formal assessment, all samples were assigned new, randomly generated identity numbers in Microsoft Excel so analyses could be performed blinded to the original fish identity.

Because selection was based partly on histological suitability and the apparent presence or absence of gill changes, the final material is not a representative random sample of the source populations. It was selected to span a range of morphologies and to support exploratory assessment of gene-morphology relationships. Consequently, the observed frequencies and severity distributions of gill changes should not be interpreted as population prevalence estimates or as a population-level normal baseline, and the estimated associations apply to the selected material.

Supplementary Fig. S2 summarizes the derivation of the linked dataset, marker completeness, and model-specific complete-case sample sizes.

### 2.2 Histological assessment

Using a recently developed semiquantitative scoring system that assesses mild-to-moderate morphological changes in gill tissue [23], the samples were examined for seven morphological changes, yielding an aggregate (sum) score for each change. The gill changes were as follows: Mucus cell number, lamellar clubbing, lamellar hypertrophy/hyperplasia, interlamellar hypercellularity, aneurysms, lamellar inflammation, and degenerative processes. The scoring method required gill filaments suitable for histological assessment, accepting slides with at least five scoreable filaments, with a target of ten. The evaluation was performed on digital slides, and slide quality was evaluated as satisfactory or not, as suboptimal quality increases the risk of misinterpretation. Fourteen gills were considered unsuitable for assessment because they had fewer than five suitable filaments or poor slide quality. The scoring was performed in a blinded manner by the same person for 1 month using digital imaging software (NDP.view2; Hamamatsu Photonics) and a customized scoring form in Excel.

### 2.3 Real-time PCR analysis

#### 2.3.1 RNA extraction

Total RNA was extracted from gill tissue according to standardized protocols, which involved trimming filaments, transferring them to lysis buffer (Qiagen RLT buffer with added DTT), and then homogenizing and centrifuging to isolate the RNA in the supernatant. After a storage period at −20°C, the automated purification process was performed using the QIAsymphony SP instrument and QIAsymphony RNA Kit (Qiagen, Hilden, Germany). The amount of gill tissue required for the process was initially determined by testing three samples according to the QIAsymphony manual. The RNA concentration was determined using a spectrophotometer (BioTek Epoch microplate spectrophotometer, Agilent Technologies). The RNA was stored at −80°C until further use.

#### 2.3.2 Genes and agents

A selection of genes essential for cell/gill function and opportunistic pathogens known to be able to reside in gill tissue without resulting in clinical gill disease were chosen (Table 2). Gene expression and the presence of most pathogens were measured in gill tissue from both freshwater and saltwater environments. As Facility 1 used freshwater with added saltwater to a salinity of 0.5-2 ppt for operational reasons, fish from this facility were also analyzed for the presence of *Paranucleospora theridion*/*Desmozoon lepeophtherii*. *Saprolegnia* spp. was only tested for gill tissue from fish in freshwater/Facility 1. A two-step RT-qPCR process was performed to produce cDNA samples, followed by two parallel gene analyses per sample, yielding an average replicate Cq value per sample (one parallel analysis for infectious agents).

**Table 2:** Gene details.

Genes	Category^a^	Sequence	Accession no.	Reference
Elongation factor 1αb (EF1αb)	Reference	EF1ab-F TGCCCCTCCAGGATGTCTAC EF1ab-R CACGGCCCACAGGTACTG	XM_014141923	
Transforming growth factor-β (TGF-β)	Immuno-logical	TGFb-F GCCATCCGTGGACAGATACT TGFb-R TCTCCCTCCTGGTCAATCTCT	XM_014129261	[19]
Interleukin-10 (IL-10)		IL10-F GGGTGTCACGCTATGGACAG IL10-R TGTTTCCGATGGAGTCGATG	XM_014168417	[19]
Interleukin-4/13α (IL-4/13α)		IL4/13a-F GCATCGTTGTGAAGAGCCAAGA IL4/13a-R GAAGTCTCCTCAGCTCCACCT	NM_001204895	[19]
Immunoglobulin T (IgT)		IgT-F CAACACTGACTGGAACAACAAGGT IgT-R CGTCAGCGGTTCTGTTTTGGA	GQ907003	[26]
Immunoglobulin M (IgM)		IgM-F TGAGGAGAACTGTGGGCTACACT IgM-R TGTTAATGACCACTGAATGTGCAT	Y12457	[26]
Interleukin-1β (IL-1β)		IL1b-F TGAAGTCCATCAGCCAGCAG IL1b-R GGATGGTGAAGGTGGTGAGG	XM_014170479	[19]
Interleukin-8 (IL-8)		IL8-F GAATGTCAGCCAGCCTTGTC IL8-R TCCAGACAAATCTCCTGACCG	NM_001140710	[27]
Cyclooxygenase-2b (Cox-2b)		Cox2b-F GGTGCTGGATGGAGAGGTGTAC Cox2b-R GGCCGAACGCCTCATG	XM_014123554	[28]
Interferon-α (IFN-α)		IFNa-F TGGGAGGAGATATCACAAAGC IFNa-R TCCCAGGTGACAGATTTCAT	AY216594	[29]
Interferon-γ (IFN-γ)		IFNg-F CTAAAGAAGGACAACCGCAG IFNg-R CACCGTTAGAGGGAGAAATG	AY795563	[29]
Serum amyloid A-5 (SAA-5)		SAA5-F GGTGAAGCTGCTCGAGGTGC SAA5-R CCATCTCCCGGCCATTACTGAT	NM_001146565	[27]
Na+,K+–ATPase α-1a (NKA α-1a)	Physio-logical	NKAa1a-F CCAGGATCACTCAATGTCACTCT NKAa1a-R GCTATCAAAGGCAAATGAGTTTAATATCATTGTAAAA	XM_014194628	[30]
Na+,K+–ATPase α-1b (NKA α-1b)		NKAa1b-F GCTACATCTCAACCAACAACATTACAC NKAa1b-R TGCAGCTGAGTGCACCAT	XM_014152156	[30]
Rhesus glycoprotein 1 (Rhcg1)		Rhcg1-F CATCCTCAGCCTCATACATGC Rhcg1-R TGAATGACAGACGGAGCCAATC	DQ431244	[31]
Opportunistic pathogens	Category^a^	Sequence	Accession no.	Reference
*Ca.* Branchiomonas cysticola (*Ca.* Branchiomonas cysticola)	Freshwater and saltwater	Bcysticola-F AATACATCGGAACGTGTCTAGTG Bcysticola-R GCCATCAGCCGCTCATGTG	OL314810	[32]
*Ichthyobodo* spp.		Ichthyobodo-F ACGAACTTATGCGAAGGCA Ichthyobodo-R TGAGTATTCACTYCCGATCCAT	MN259577	[33]
*Paranucleospora theridion*		Ptheridion-F CGGACAGGGAGCATGGTATAG Ptheridion-R GGTCCAGGTTGGGTCTTGAG	KR187185	[34]
*Saprolegnia* spp.	Freshwater	Saprolegnia-F GGDGCTCCWGATATGGCTTTNCC Saprolegnia-R CTGAAGGACCWGAGTGHGCTTG	OK872247	[35]

a Genes have multiple functions, but the category listed was the function-/group variable used in the statistical analyses. The category listed for opportunistic pathogens reflects gill analysis performed in a specific RAS environment: freshwater (Facility 1) or saltwater (Facility 2).

For each gill sample, 1000 ng of RNA was used for cDNA synthesis with a combination of anchored oligo(dT) and random hexamer primers, following the Transcriptor First Strand cDNA Synthesis Kit (Agilent Technologies, Matriks AS, Oslo, Norway) protocol. The synthesized cDNA was diluted 5 times by adding 80 μl of RNase-free water and stored at −20°C until further use.

Quantitative real-time PCR (qPCR) was performed using the QuantiTect SYBR Green PCR Kit (Qiagen, Germany) on an AriaMx Real-Time PCR System (Agilent Technologies), according to the manufacturer's instructions. For each reaction, 1.0 μl of diluted cDNA (prepared from 1000 ng of total RNA) was used as a template in a 20 μl total reaction volume containing 10 μl of 2× QuantiTect SYBR Green Master Mix, 1 μl of each host gene-specific primer (10 μM), and 7 μl of RNase-free water. Assay efficiency for the host genes was determined for each primer pair using 10-fold serial dilutions of pooled cDNA, run in duplicate. Target efficiencies between 90% and 110% were accepted. For the Rhcg1 target, efficiency exceeded this range slightly (113%).

Testing for *Ca.* Branchiomonas cysticola and *Paranucleospora theridion* were performed using validated commercial kits (Aquakit, SBC-Biotech, Taiwan), which included a positive control.

Thermal cycling conditions consisted of an initial activation step at 95°C for 15 min, followed by 40 amplification cycles of denaturation at 95°C for 15 s, annealing at 57°C for 30 s, and extension at 72°C for 30 s. The specificity of the amplified products was verified by melting curve analysis and agarose gel electrophoresis.

Host gene expression was normalized to the reference gene EF1αb, and primer efficiency correction was applied before calculating ΔCq values as: ΔCq = Cq_target − Cq_reference.

Samples with a replicate ΔCq > 1.5 were flagged, and the most consistent value was retained if replicates diverged substantially. Samples lacking amplification of the reference gene (n = 2) were excluded from further analysis. In cases with only one successful replicate, the available value was retained.

EF1αb was selected as the reference gene based on prior validation in Atlantic salmon [36]. Its raw Cq was stable across the sample set (mean 15.84, SD 0.66, coefficient of variation 4.2%; 98% of samples within ±1.5 Cq of the median) and did not differ significantly between freshwater (15.98 ± 0.70; mean ± SD) and saltwater (15.74 ± 0.62) fish (median difference 0.25 Cq; Mann–Whitney p = 0.068), supporting its use as a single normaliser (Supplementary Fig. S1). Duplicate-well reproducibility was high (median difference 0.36 Cq). A single reference gene does not, however, permit calculation of a formal stability index (e.g., geNorm M/NormFinder), and inclusion of a second reference gene would be preferable in future studies.

### 2.4 Statistical analysis

Statistical analyses were conducted using Stata 16, Stata 18, and RStudio (version 2026.01.2; packages: MASS, car, ggplot2, pscl). Morphological gill changes were evaluated using aggregate scores for each of the seven histological parameters. Descriptive statistics summarized morphological, gene expression, and pathogen load data across sampling environments and fish groups. Given the non-normal distribution of gene expression data (as assessed via Shapiro–Wilk tests and visualized using kernel density and normal quantile plots), nonparametric tests were employed for group comparisons. We used the Wilcoxon rank-sum test to compare two groups (e.g., freshwater vs. saltwater environments), whereas the Kruskal–Wallis test compared groups within each environment.

Associations between gene expression and gill histological scores were examined using generalized linear models. Negative binomial regression was used for count-based morphological scores (e.g., mucous cell proliferation, lamellar clubbing, lamellar inflammation) when overdispersion was detected (confirmed via deviance and dispersion statistics). Poisson regression was applied when the variance equals the mean.

Linear regression was employed for continuous outcome variables when the residuals were homoscedastic (as assessed by the Breusch–Pagan/Cook–Weisberg test). Collinearity diagnostics guided model selection. Variance Inflation Factors (VIFs) were calculated to assess multicollinearity among predictors, and variables with VIFs greater than 5 were systematically excluded or grouped to reduce redundancy. We also performed pairwise correlation analyses among predictors to inform model refinement. Model fit was assessed using likelihood ratio tests, pseudo-R^2^ (for count models), and adjusted R^2^ (for linear models). Regression coefficients were reported alongside 95% confidence intervals and p-values. Scatterplots and kernel density plots were generated to visualize key gene–morphology relationships for straightforward interpretation. Depending on the model context, ΔCq values were used as either continuous predictors or response variables and were interpreted inversely (i.e., lower ΔCq values indicated higher expression).

For clarity, results are described in terms of expression throughout: because ΔCq is inversely related to expression, a negative regression coefficient on ΔCq denotes a positive association between transcript level and the histological score. To account for multiple comparisons, p-values from the final association models were adjusted using the Benjamini–Hochberg procedure (false-discovery rate = 0.05; Supplementary Table S1). Because these models were reached through prior collinearity-based variable selection, the correction is applied to the final models and interpreted as approximate. Given the number of markers, scores, and subgroup comparisons relative to the sample size, the association analyses are regarded as exploratory and hypothesis-generating.

### 2.5 Ethics statement

Ethical approval was not required for the studies involving animals, in accordance with local legislation and institutional requirements, because the Food Inspection Agency does not require ethical approval for sampling material from fish held in normal or standard production environments. Written consent was obtained from the fish owners (the farm operators) for the use of the sampled material.

## 3 Results

Results are from analyses of 111 fish with histological and PCR-linked data.

### 3.1 Histological scores

Within the selected sample of apparently healthy salmon, mild gill changes were observed across both production systems and sampling time points (Fig. 1). The observed frequencies describe the selected material and should not be interpreted as estimates of population prevalence.

**Fig. 1:**
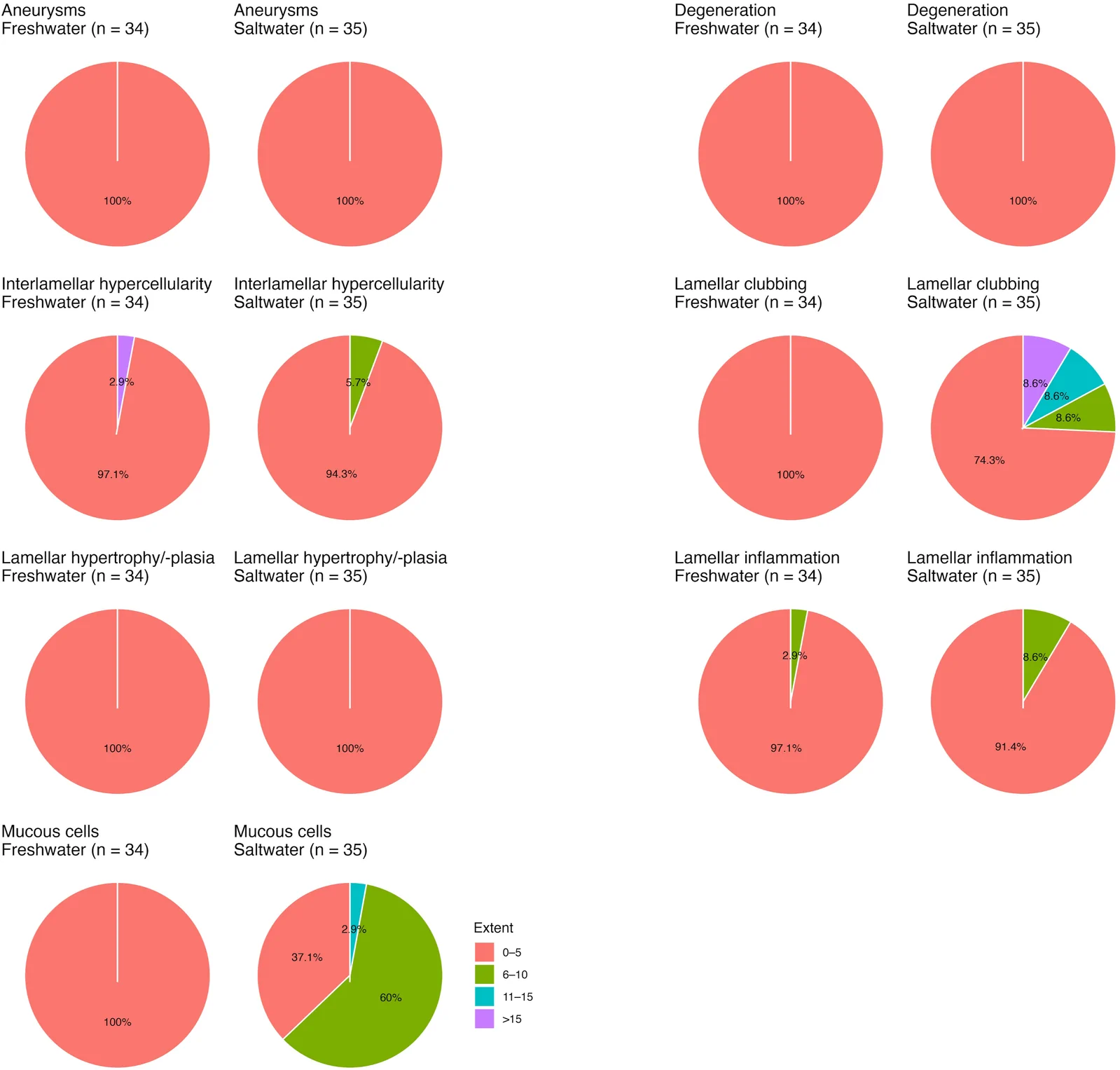
Diagrams showing the distribution of gill changes across environments.

Within the selected material, increased mucous cell numbers (Fig. 2a) and lamellar clubbing (Fig. 2b and c) were more prominent among fish from the saltwater production system (Facility 2), and lamellar inflammation scores were also higher (Fig. 1). Because the saltwater production system also differed from the freshwater production system (Facility 1) in facility, fish size, life stage, and sampling time, these differences cannot be attributed to water type alone. The other histological parameters were more similar between the two systems. Within the saltwater production system, lamellar clubbing scores were higher in FG4 than in FG3.

**Fig. 2:**
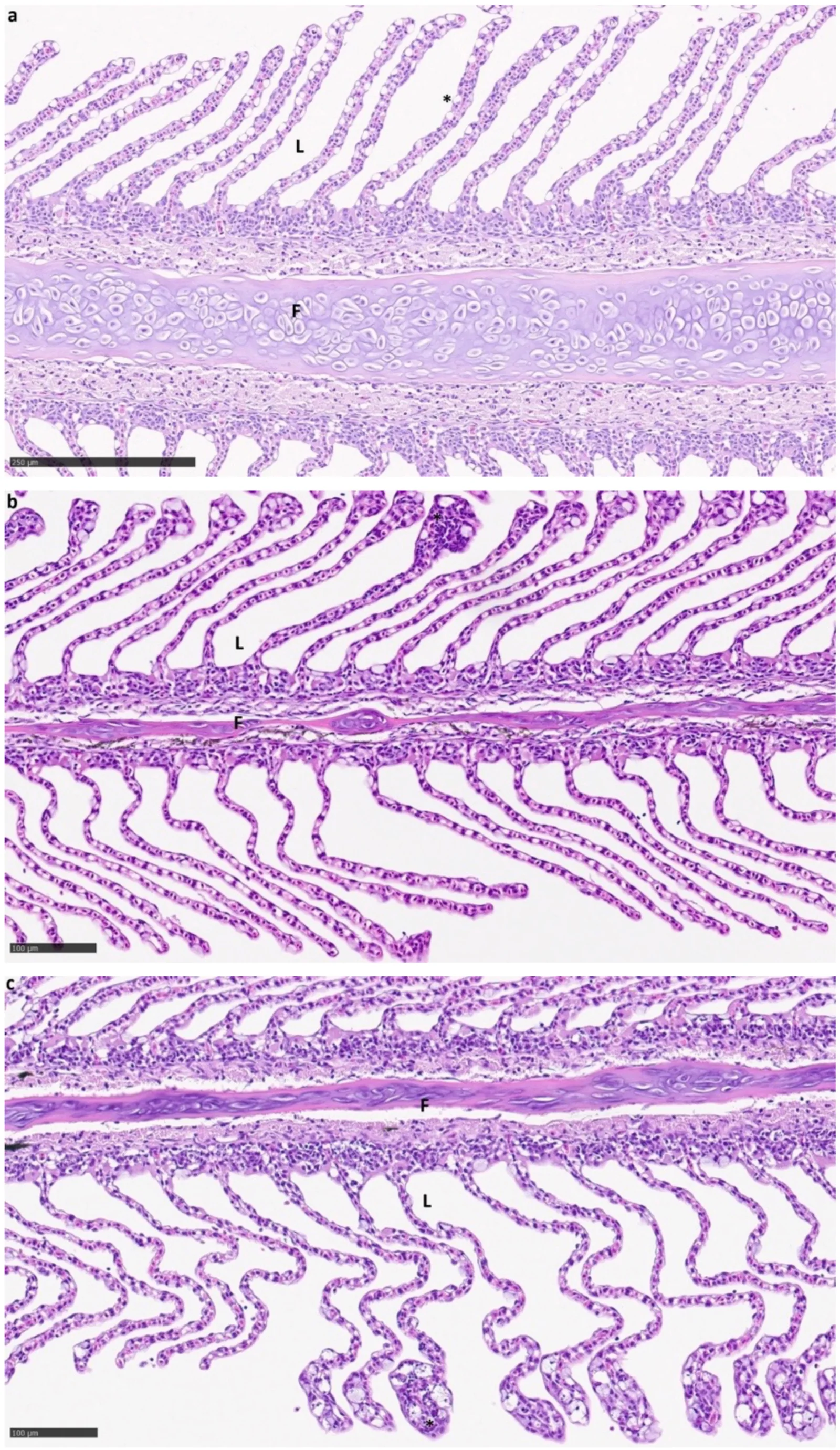
Examples of mucous cells (**a**) and lamellar clubbing with inflammation and degeneration (**b/c**) are denoted with an asterisk (*). F=Filament, L = Lamella.

The histological scores of fish classified as deviant did not differ notably from those of apparently healthy fish within the saltwater production system, except for a tendency towards higher interlamellar hypercellularity and degeneration scores (Fig. 3).

**Fig. 3:**
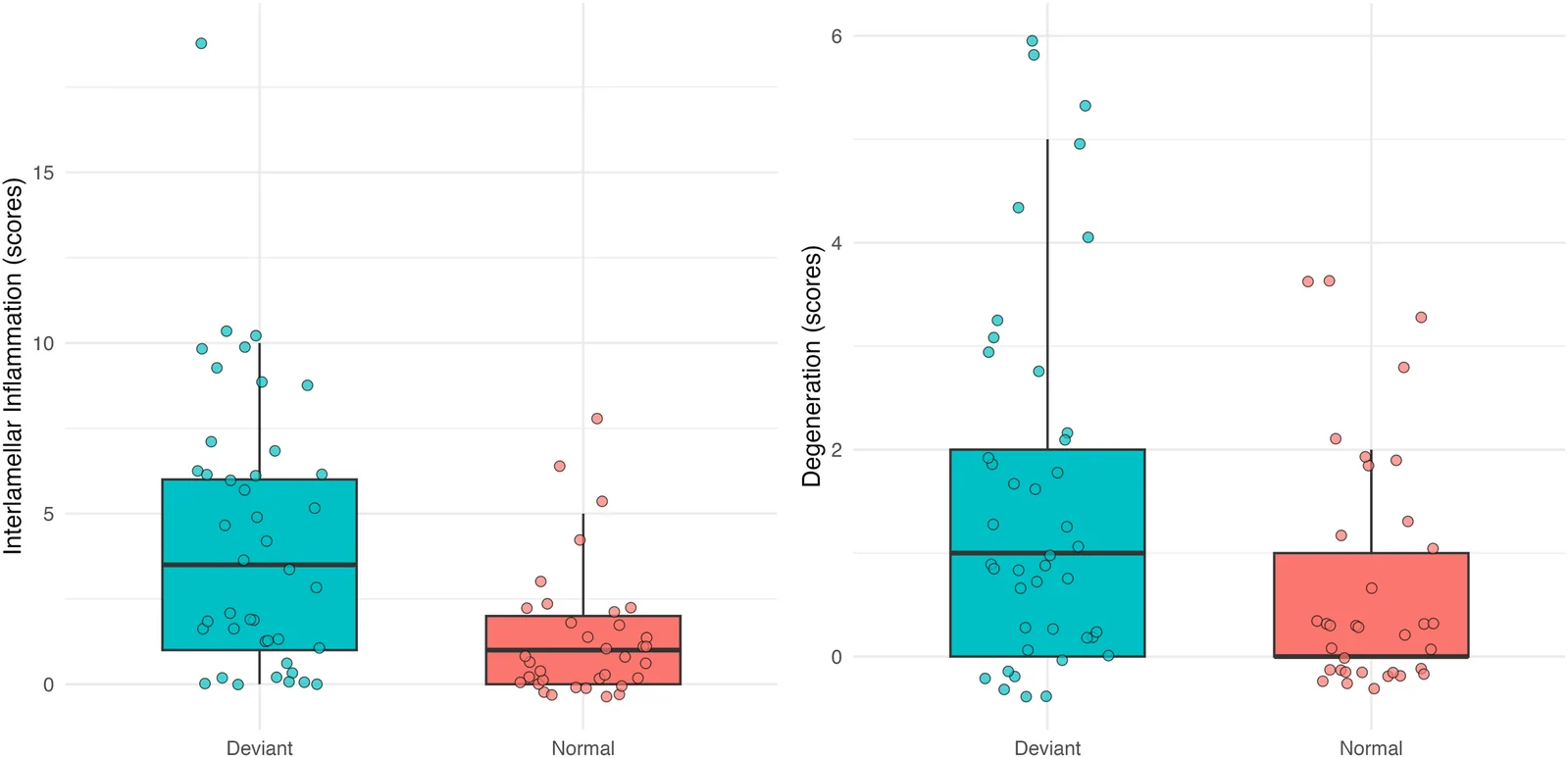
Comparison box plots between apparently healthy and deviant fish from the saltwater production system for two categories of gill changes.

### 3.2 Immunological and physiological markers

Kernel density plots documented bimodality for several immunological and physiological markers (Fig. 4). Normal quantile plots also showed the same pattern (Fig. 5), and the Shapiro-Wilk assessment indicated a violation of normality.

**Fig. 4:**
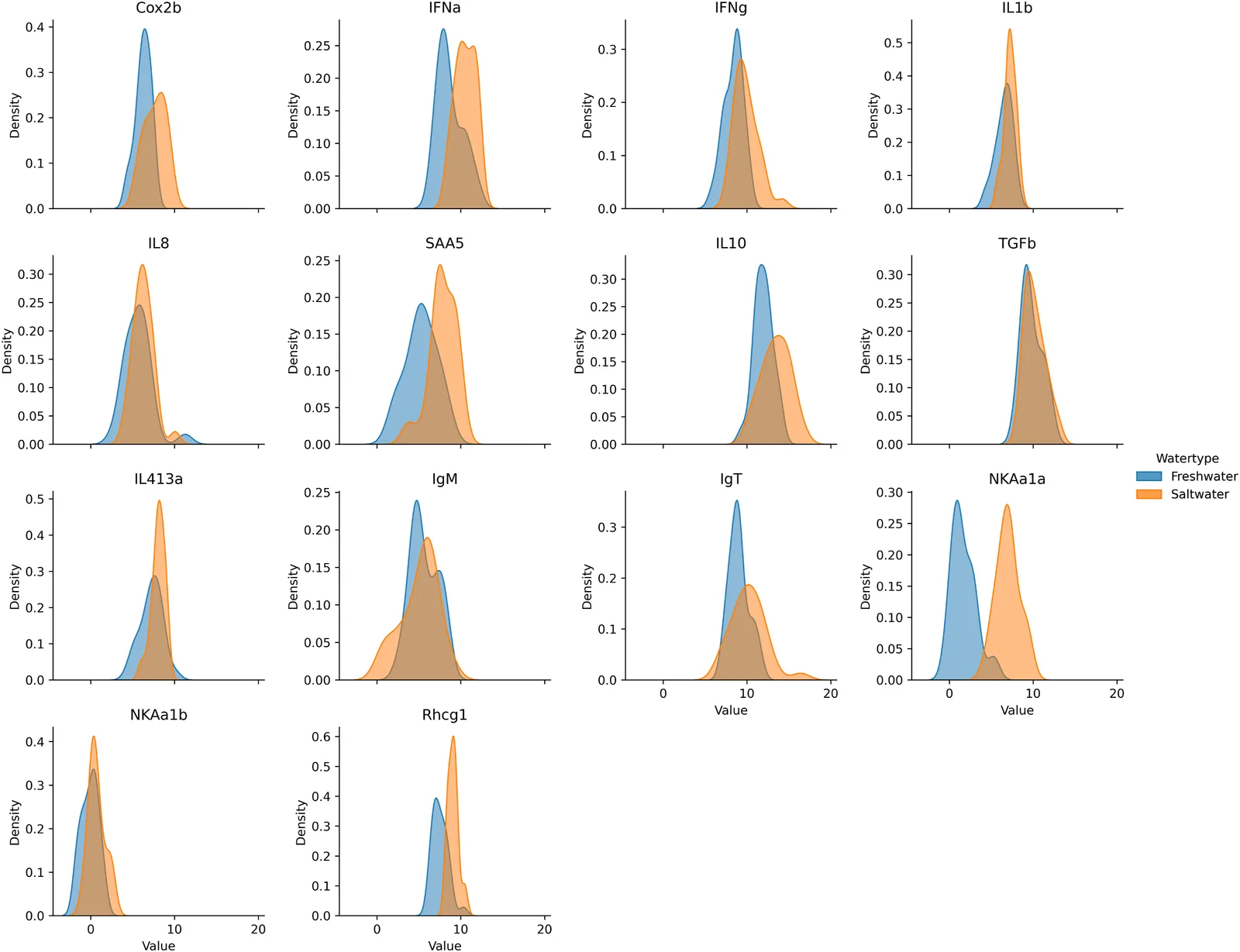
Kernel density plots for each gene.

**Fig. 5:**
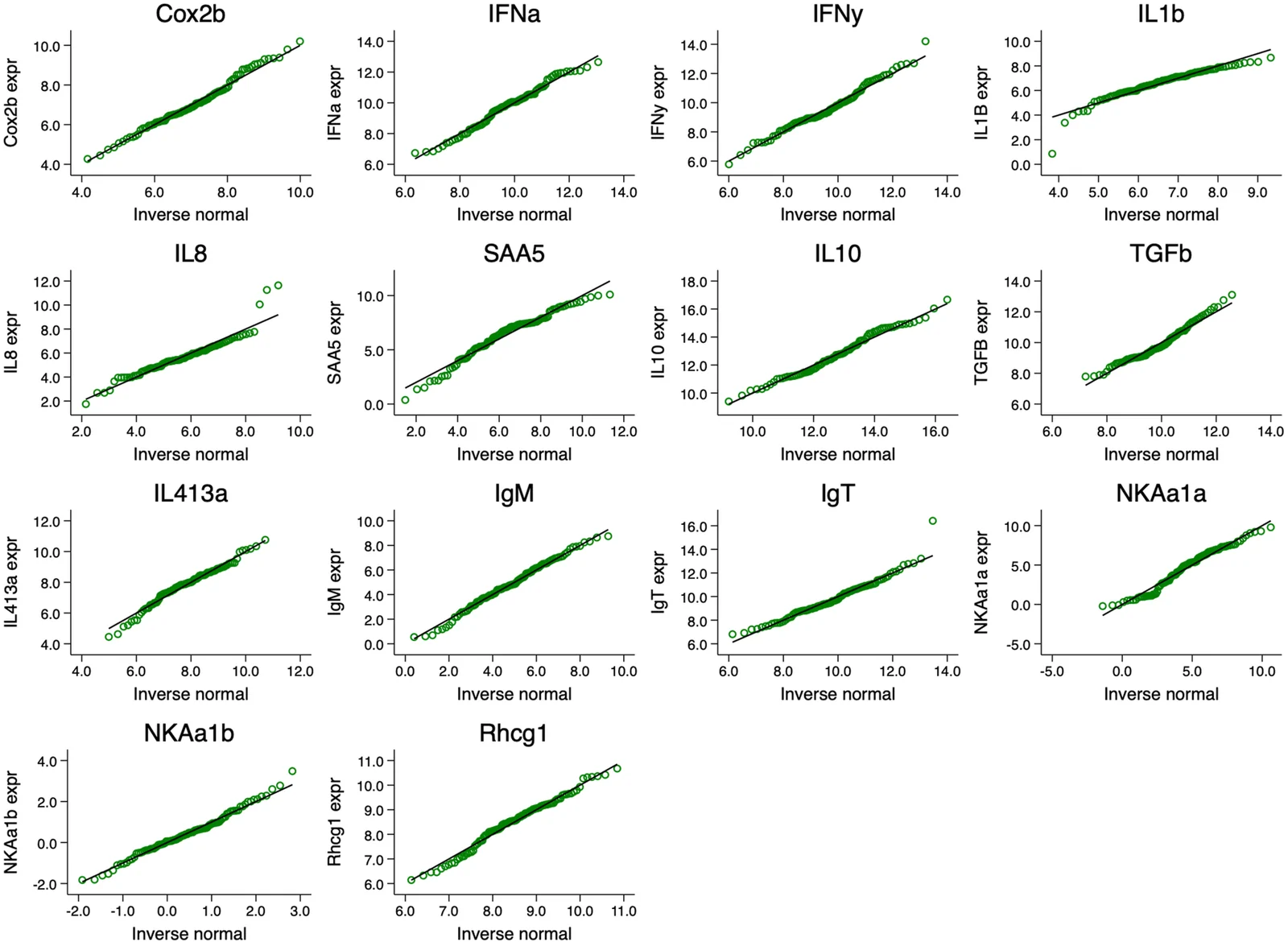
Normal quantile plots for each gene.

Gene expression levels were generally higher in fish kept in freshwater (lower ΔCq) than in saltwater (higher ΔCq) environments (Fig. 6). Exceptions were IL-8, IgM, and TGF-β, which appeared relatively similar across the two environments.

**Fig. 6:**
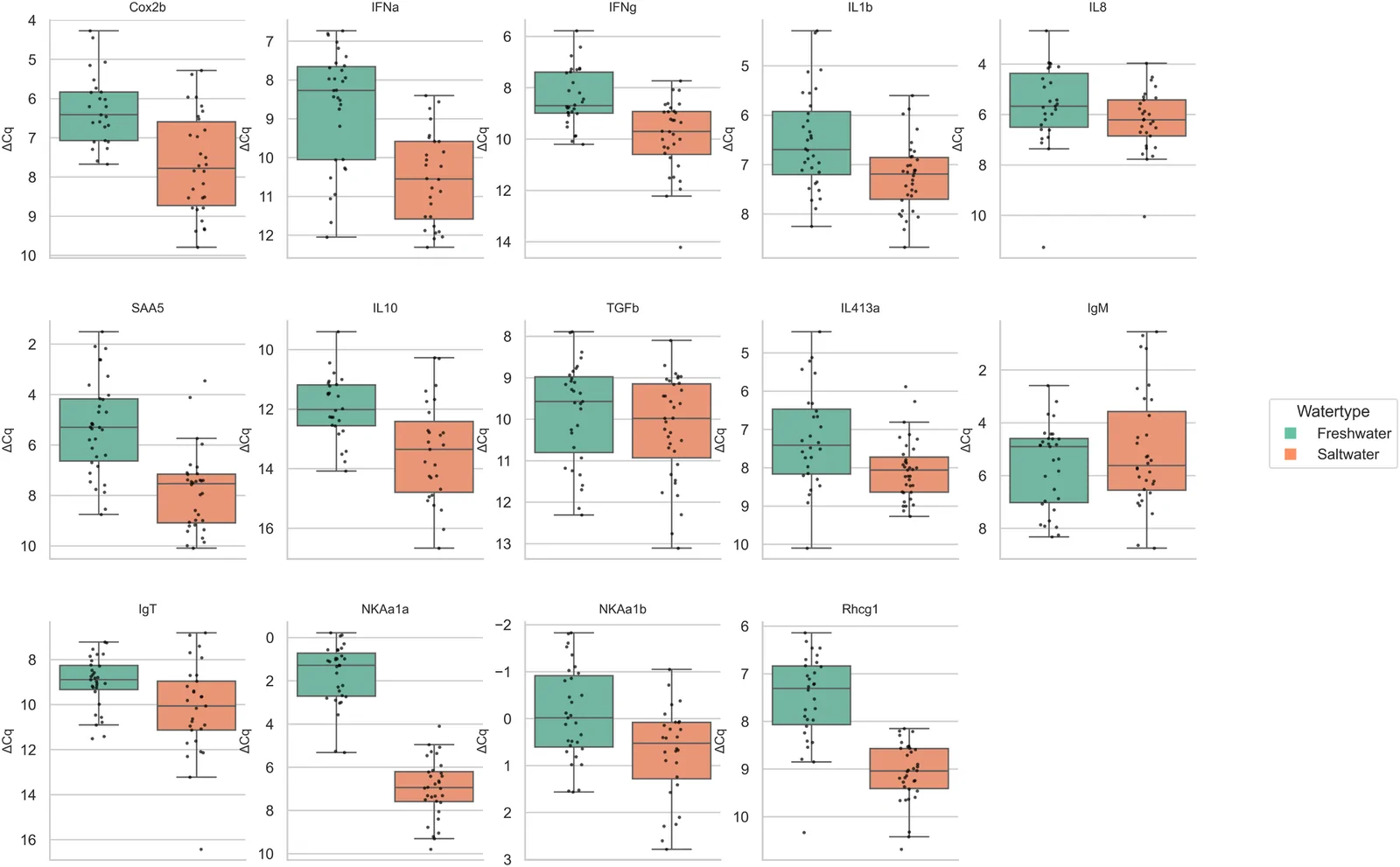
Box plots showing relative gene expression (ΔCq values) of selected immunological and physiological markers in apparently healthy Atlantic salmon from the freshwater and saltwater production systems. Note that the y-axis is inverted; lower ΔCq values indicate higher relative gene expression.

This agreed with the two-sample Wilcoxon rank-sum (Mann-Whitney) test. Furthermore, the Kruskal-Wallis equality-of-populations rank test revealed differences between fish groups within environments, except for IgM.

The environmental differences were most pronounced for IFN-α, SAA-5, NKA α-1a, and Rhcg1 (Fig. 6). NKA α-1a expression was highest in fish residing in freshwater, and NKA α-1b was higher than NKA α-1a in saltwater fish. At each sampling time point, the α-1b expression levels in freshwater were generally higher than α-1a, except for the smallest fish, and both α-1b (in freshwater) and α-1a (in saltwater) were detectable and expressed relatively similarly across fish sizes. There were differences between apparently healthy and deviant fish in saltwater for IL-1β, IL-8, SAA-5, IgM, and NKA α-1a (Fig. 7).

**Fig. 7:**
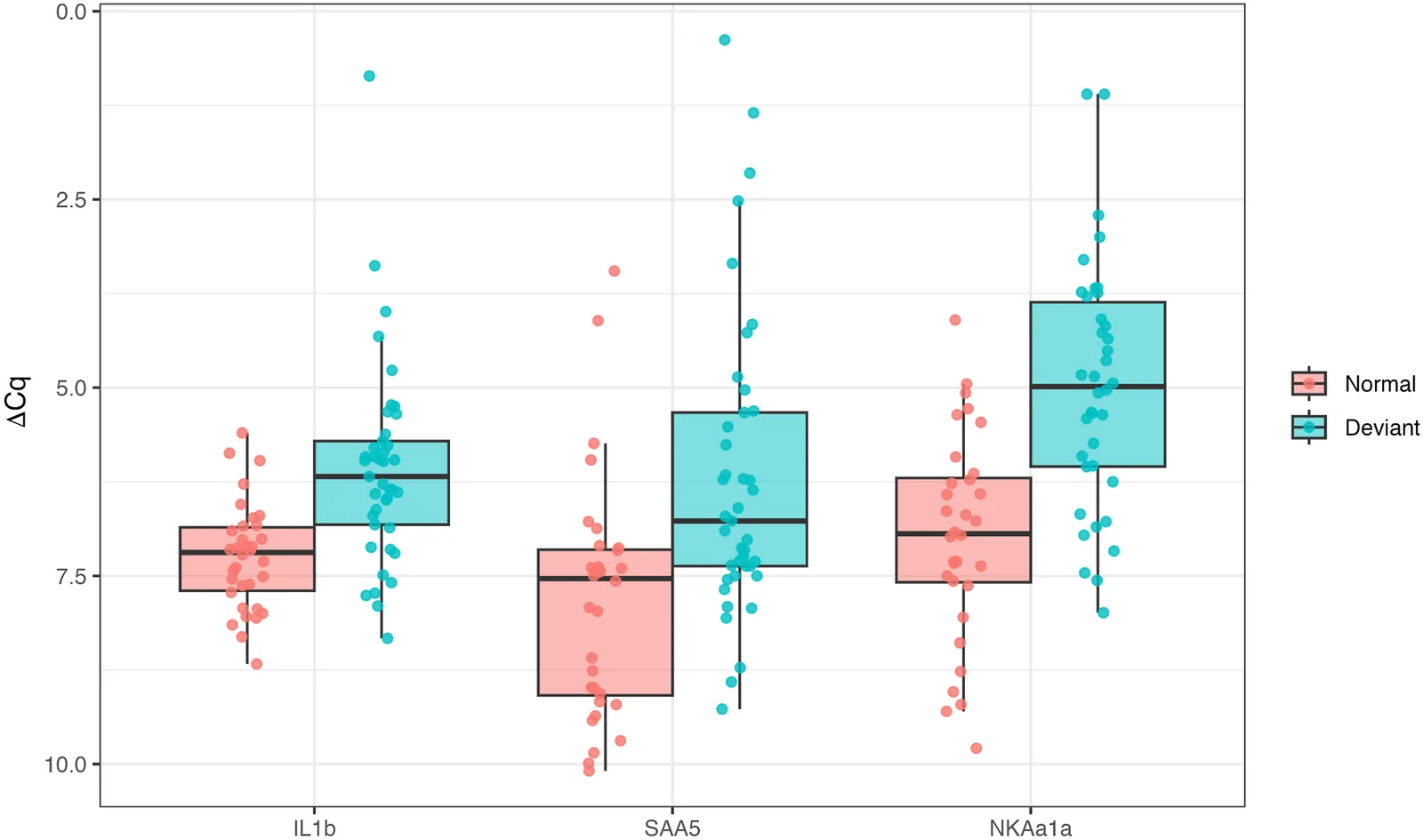
The difference in gene expression between apparently healthy and deviant fish in saltwater (lower ΔCq means higher expression).

### 3.3 Gill opportunistic pathogens

Analyses for *Ichthyobodo* spp., *Paranucleospora theridion,* and *Saprolegnia* spp. were negative. *Ca.* Branchiomonas cysticola was found in fish group 2 (9 of 23 positive fish, average Cq 34, range 27-38) and fish group 4 (15 of 16 positive, average Cq 29 and range 23-37) but not in fish group 1 and 3. *Ca.* Branchiomonas cysticola loadings appeared to increase over time and correlated positively with most of the immunological markers assayed, indicating increased expression. In saltwater, higher loadings were associated with increased IgT and IgM expression and reduced IL-4/IL-13α expression. Higher Ca. Branchiomonas cysticola loadings coincided with increased lamellar inflammation aggregate scores, most evident in saltwater (Fig. 8). These relationships are associative and, in this cross-sectional material, do not establish a causal role in gill pathology.

**Fig. 8:**
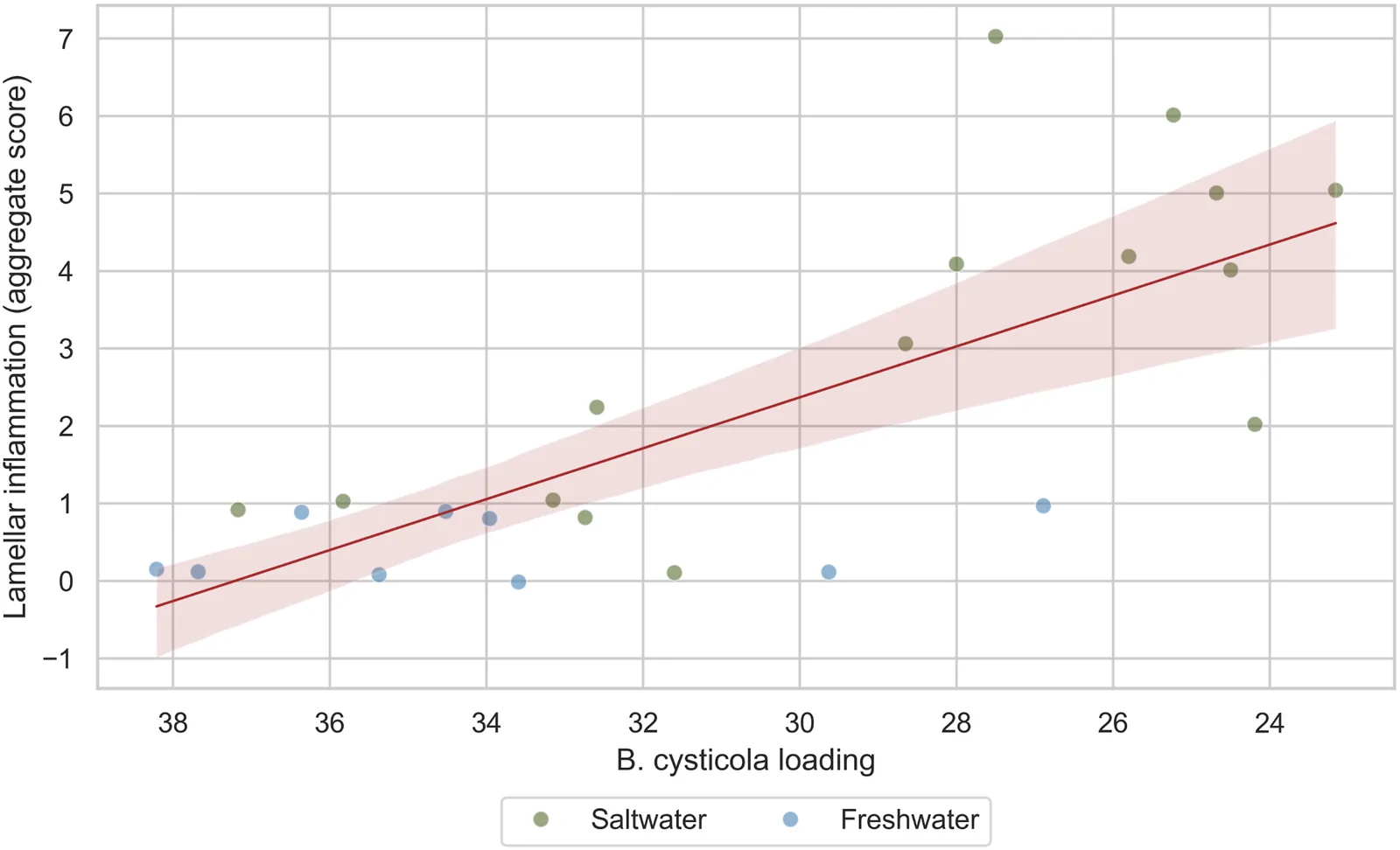
Scatterplot of Ca. Branchiomonas cysticola and lamellar inflammation score with 95% CI.

### 3.4 Expression of immunological and physiological markers against gill morphology

Our initial analyses examined associations between gene expression and gill changes within the selected apparently healthy material. We focused on mucous cell proliferation, lamellar clubbing, and lamellar inflammation because these were the most distinct changes represented among fish from the saltwater production system (Fig. 1).

#### 3.4.1 Immunological markers

Mucous cell scores were plotted against the immunological markers (Fig. 9), with no apparent correlation between mucous cell scores and different immune markers. In contrast, several of the markers were correlated (collinearity).

**Fig. 9:**
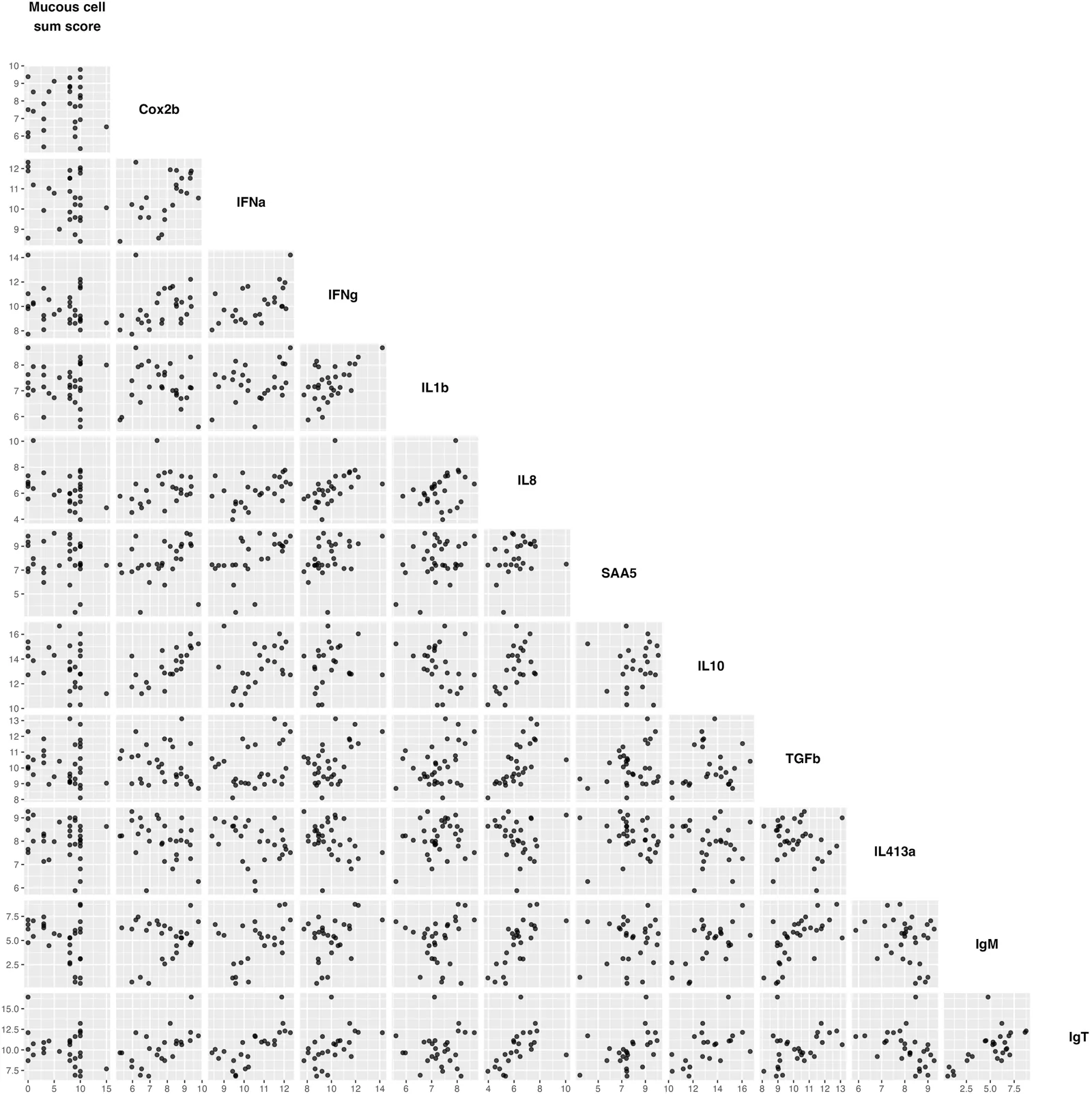
Scatterplot matrix showing correlations of gene expression (x-axis, lower ΔCq means higher expression) with mucous cell numbers (y-axis) in saltwater. Deviant fish are omitted.

We then analyzed relationships among multiple predictors. The response variables, mucous cell counts, lamellar clubbing, and lamellar inflammation scores, were assessed against the comprehensive set of predictors (markers) in regression models. As expected, when many variables were included simultaneously, multicollinearity emerged, leading to instability in coefficient estimates, inflated standard errors, and reduced statistical power. This phenomenon makes it challenging to determine the independent effect of each predictor on the response variable, resulting in misleading or insignificant p-values even when genuine relationships exist [37,38]. The variance inflation factor (VIF) was calculated for each predictor using the response variable to diagnose multicollinearity. VIF measures the extent to which the variance of a regression coefficient is inflated due to multicollinearity with other predictors. As a rule, VIF values exceeding 5 or 10 indicate problematic multicollinearity that can adversely affect the model's reliability [38,39]. In the initial models that included all predictors, VIF values often exceeded these thresholds, suggesting severe collinearity. The issue of multicollinearity was addressed by systematically testing smaller groups of predictors across different rounds of regression analysis. This approach allowed the identification of specific variables that correlated with others and contributed most to the multicollinearity problem. By reducing the number of predictors and evaluating variable subsets independently, models with acceptable VIF values (typically below 5) were achieved. Predictors that consistently exhibited high VIFs were either removed or combined with other predictors to reduce redundancy.

In addition to evaluating VIF, pairwise correlations between predictors were calculated to identify potential dependencies. This process was essential for determining which predictors could be safely included in the same model without inducing collinearity. Furthermore, to enhance robustness and comparability, the effects of predictors were assessed using both standard linear regression, Poisson regression, and negative binomial regression models. The latter was relevant when evidence of overdispersion was present in the response variables. This iterative process of model simplification and refinement enabled the identification of combinations of statistically significant and biologically relevant predictors without being compromised by multicollinearity. A clearer understanding of their independent contributions to the different dependent variables was achieved by strategically reducing the number of predictors in each model.

##### 3.4.1.1 Mucous cells

Before FDR correction, Cox-2b, IgM, and IL-4/13α showed nominal associations with mucous cell scores (Table 3, Fig. 10). After correction, only the association with IgM remained significant (Supplementary Table 1). This indicates that mucous scores increase as IgM and IL-4/13α levels increase (a negative value indicates declining Cq values). For Cox-2b, the overall coefficient was 0.22 but varied over the range of Cq values (Fig. 10). When expression increased, the mucous cell scores dropped.

**Table 3:** The results from the negative binomial analysis using Cox-2b, IgM, and IL-4/13α as predictors of mucous cell scores are shown before FDR correction (n = 28).

Term	Estimate	Std. Error	95% CI Lower	95% CI Upper	p-value	
(Intercept)	3.584	1.677	0.312503517	6.91211713	0.0326	*
Cox-2b	0.220	0.109	0.003574994	0.44058821	0.0434	*
IgM	−0.203	0.060	−0.323101942	−0.08591355	< 0.001	***
IL-4/13α	−0.321	0.149	−0.622673734	−0.02700839	0.0318	*

**Fig. 10:**
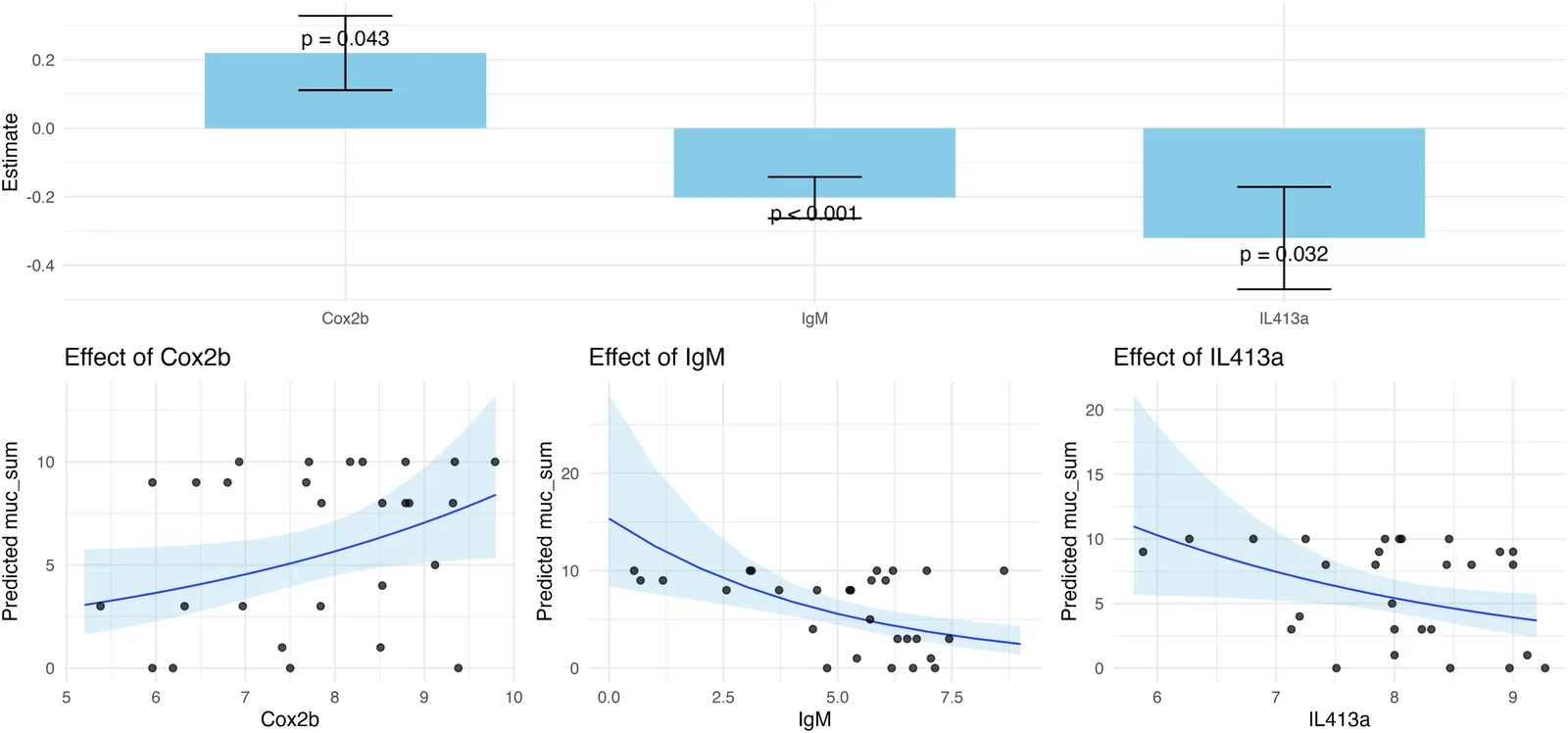
Before FDR correction, the negative binomial model showed nominal associations for Cox-2b, IgM, and IL-4/13α with mucous cell scores. Mucous scores increase as expression of Cox-2b drops (lower ΔCq means higher expression), while IgM and IL-4/13α were positively correlated with mucous cell scores.

The model was statistically significant overall (LR χ^2^(3) = 12.2, p = 0.007), with a pseudo-R^2^ of 0.077, indicating low explanatory power.

##### 3.4.1.2 Lamellar clubbing

A negative binomial regression model was employed to investigate predictors of lamellar clubbing. This analysis identified IFN-α and IgT as significant predictors (Table 4, Fig. 11).

**Table 4:** The results obtained from the negative binomial analysis for IFN-α and IgT as predictors of lamellar clubbing are shown (n = 25).

Term	Estimate	Std. Error	95% CI Lower	95% CI Upper	p-value	
(Intercept)	12.401	1.680	9.2822835	15.90115478	< 0.001	***
IFN-α	−0.878	0.177	−1.2397161	−0.54375139	< 0.001	***
IgT	−0.248	0.099	−0.4405164	−0.06369994	0.0121	*

**Fig. 11:**
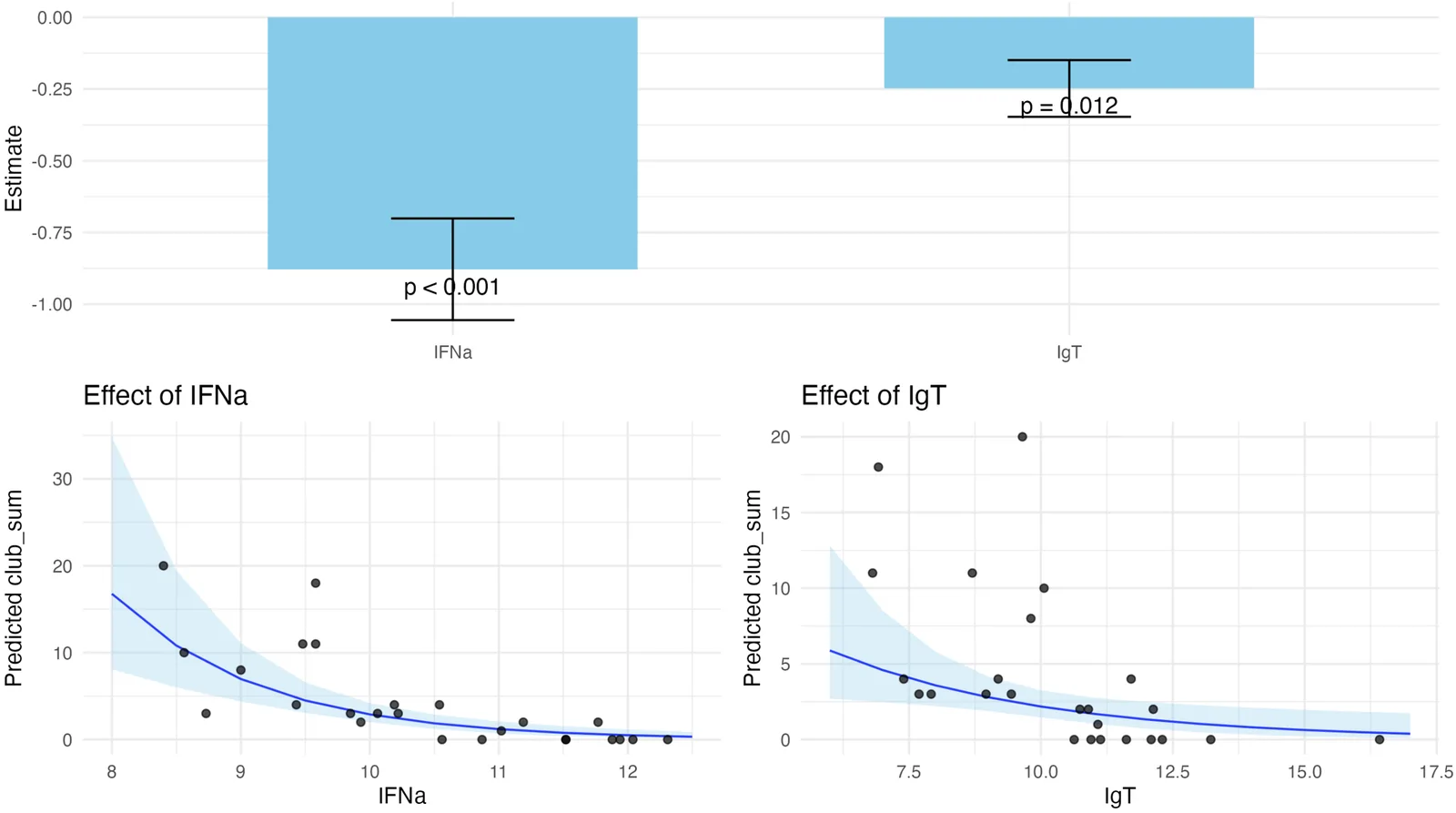
The negative binomial model revealed a significant association between IFN-α and IgT, both of which were positively correlated with lamellar clubbing (lower ΔCq, x-axis, means higher expression).

The model was statistically significant overall (LR χ^2^(2) = 19.6, p < 0.0001), with a pseudo-R^2^ = 0.247, indicating good explanatory power. IFN-α and IgT expression were positively correlated with lamellar clubbing, i.e., declining Cq-values coincided with increased lamellar clubbing score.

##### 3.4.1.3 Lamellar inflammation

To investigate associations between the predictors and the response variable, lamellar inflammation, we used negative binomial regression, an extension of Poisson regression, to account for overdispersion in the data, and IgT was the only predictor explaining significant variation in the response variable (Table 5, Fig. 12). Higher expression of IgT aligns with increased lamellar inflammation.

**Table 5:** The results obtained from the negative binomial analysis, using IgT as a predictor of lamellar inflammation, are shown (n = 29).

Term	Estimate	Std. Error	95% CI Lower	95% CI Upper	p-value	
(Intercept)	3.56	0.735	2.1402753	5.0023064	< 0.001	***
IgT	−0.29	0.078	−0.4465348	−0.1424271	< 0.001	***

**Fig. 12:**
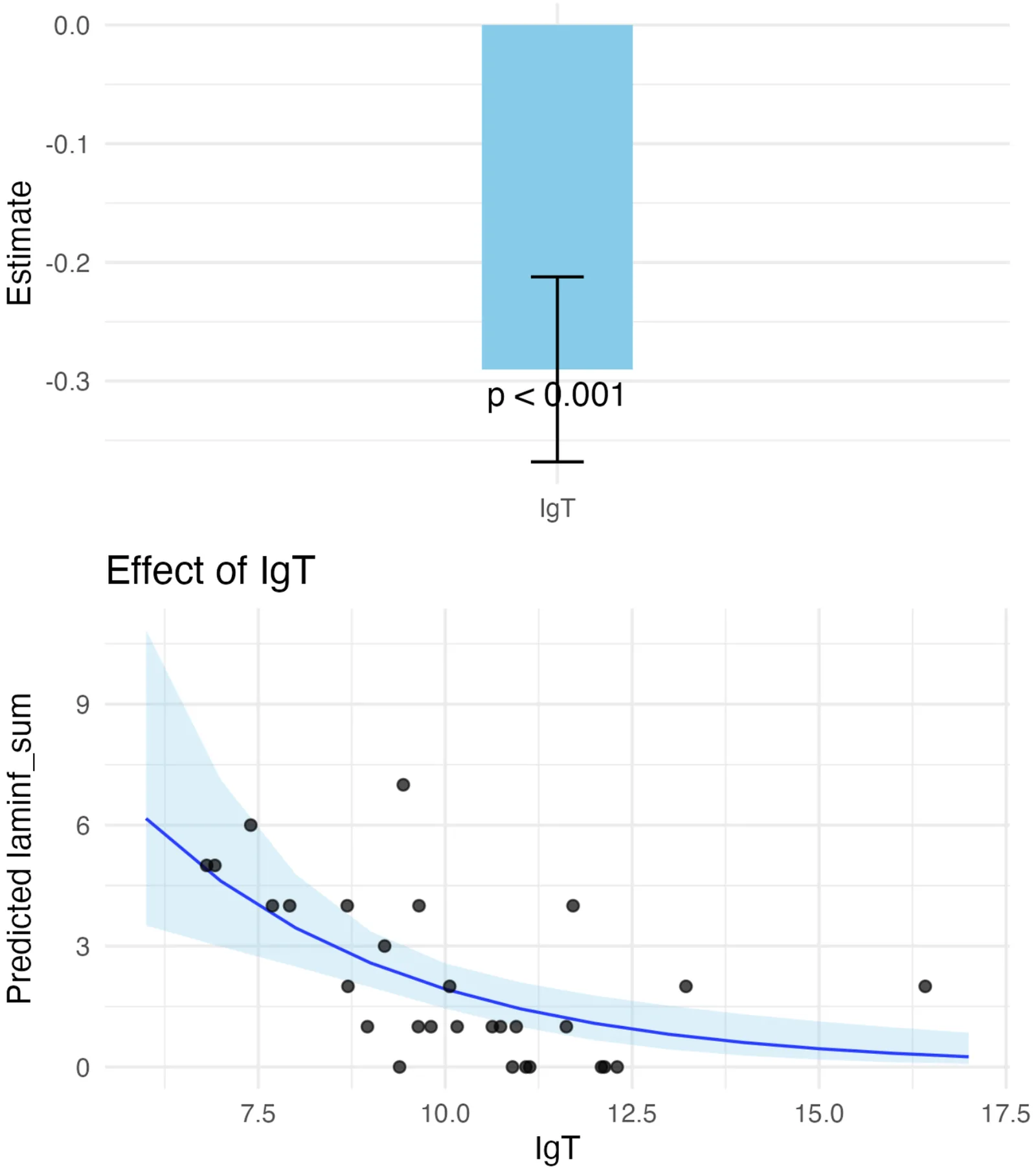
The negative binomial model revealed a significant association between IgT (lower ΔCq, x-axis, means higher expression) and lamellar inflammation.

#### 3.4.2 Physiological markers

The scatter plot for mucous cell scores against physiological markers showed a similar pattern to that for the immunological markers (Fig. 13).

**Fig. 13:**
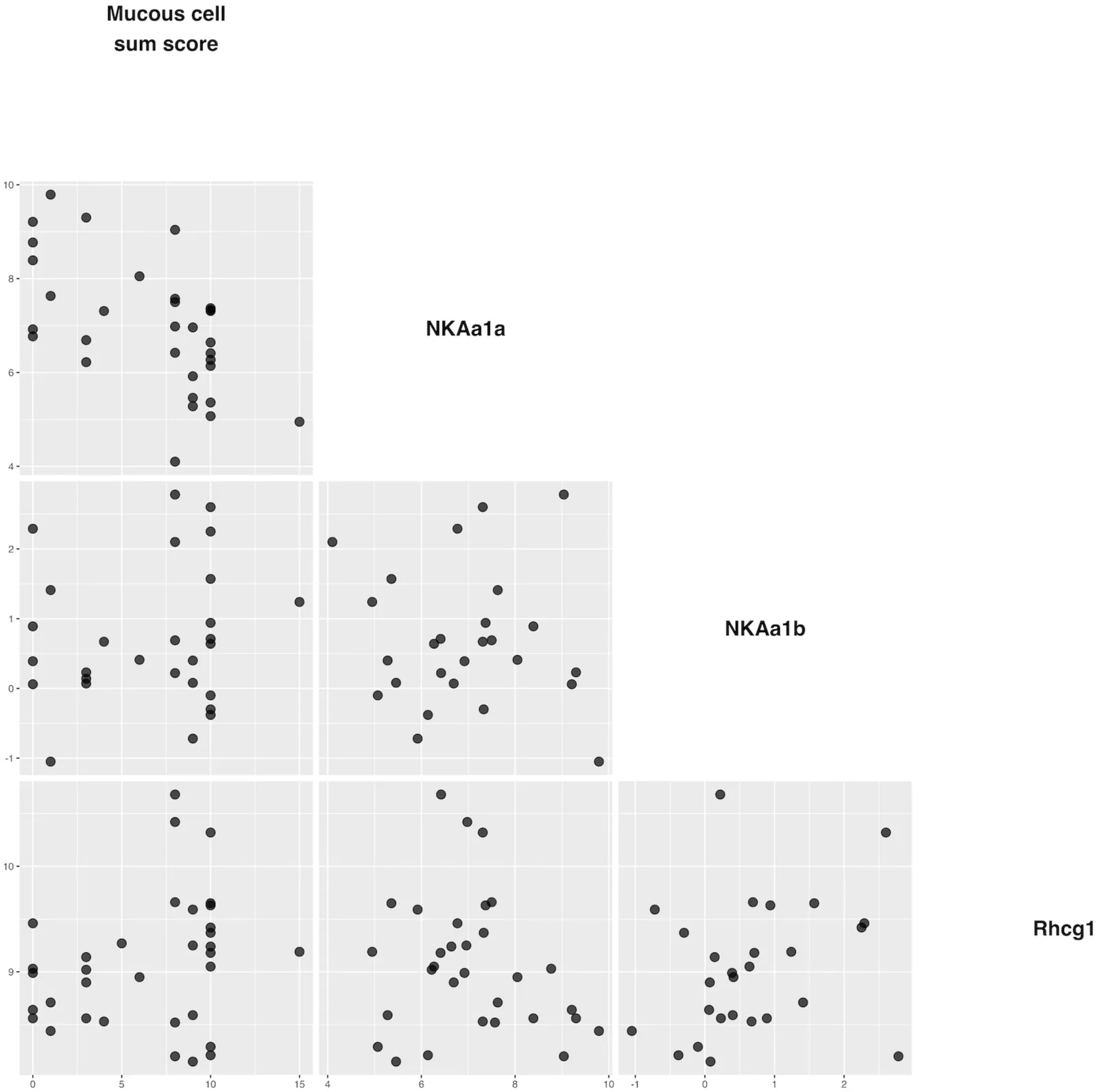
Scatterplots of physiological markers against mucous cell scores (y-axis) in apparently healthy fish from the saltwater production system; deviant fish were omitted. Note that lower ΔCq (x-axis) means higher expression.

NKA α-1a was the only significant predictor (p = 0.002) for mucous cell scores, while NKA α-1b and Rhcg1 were not significant predictors (p-values > 0.05, Table 6). The linear model was statistically significant overall, but only NKA α-1a significantly explained mucous cell scores. VIF values were <1.5, and the residual plot showed no indication of heteroskedasticity (Breusch–Pagan/Cook–Weisberg test gave p = 0.83).

**Table 6:** Results for linear regression for mucous cell scores with NKA α-1a, NKA α-1b, and Rhcg1 as predictors. NKA α-1a expression correlates positively with mucous cell scores (n = 25). Linear regression.

Mucous cell (sum)	Coef.		St.Err.	t-value		p-value	[95% Conf		Interval]		Sig
NKA α-1a	−1.909		0.539	−3.54		0.002	−3.03		−0.788		***
NKA α-1b	0.186		0.788	0.24		0.815	−1.453		1.826		
Rhcg1	0.932		1.164	0.80		0.432	−1.489		3.352		
Constant	11.468		11.769	0.97		0.341	−13.006		35.943		

Mean dependent var		6.560			SD dependent var			4.360			
R-squared		0.418			Number of obs			25			
F-test		5.021			Prob > F			0.009			
Akaike crit. (AIC)		138.028			Bayesian crit. (BIC)			142.903			
***p<0.01, **p<0.05, *p<0.1											
											

For lamellar inflammation, increased NKA α-1a expression correlated with higher scores (p = 0.034), with no indication of collinearity; the Breusch–Pagan/Cook–Weisberg test yielded p = 0.18 (no heteroskedasticity). For lamellar clubbing, none of the predictors had p-values <0.05.

After Benjamini–Hochberg correction across the final association models (Supplementary Table S1), the immunological associations of lamellar clubbing with IFN-α (q < 0.001) and IgT (q = 0.030), lamellar inflammation with IgT (q = 0.003), mucous cell numbers with IgM (q = 0.004), and mucous cell numbers with NKA α-1a (q = 0.007) remained significant. The association between lamellar inflammation and NKA α-1a, together with associations involving IL-4/13α, Cox-2b, NKA α-1b, and Rhcg1, did not remain significant. These non-surviving associations are regarded as nominal and exploratory only.

## 4 Discussion

The selected material showed mild morphological alterations in apparently healthy Atlantic salmon from both commercial RAS production systems. Within this material, scores for increased mucous cell numbers, lamellar clubbing, and lamellar inflammation were higher among fish from the saltwater production system, particularly among larger individuals. These differences are descriptive because the water environment was inseparable from facility, fish size, life stage, and sampling time. Gene expression profiles also differed between systems and fish groups. After correction for multiple testing, the most robust gene-morphology associations involved IFN-α and IgT with lamellar clubbing or inflammation, IgM with mucous cell numbers, and mucous cell numbers with NKA α-1a. Ca. *Branchiomonas cysticola* was detected at subclinical levels and coincided with increased lamellar inflammation, but the cross-sectional design does not support causal inference.

Apparently healthy gills are not necessarily free of morphological change [1]. The selected samples demonstrate that mild alterations can be present in fish without overt gill disease. However, because the material was deliberately selected to include a range of morphologies, the present study cannot determine how common these changes are in the source populations or define population-level normal gill morphology. Only a limited number of studies have systematically evaluated morphological variation in apparently healthy fish gills using histological scoring in tilapia, rainbow trout, and Atlantic salmon [[40], [41], [42]]. These studies reported epithelial hypertrophy and hyperplasia, inflammation, and mucous cell proliferation among the observed findings. The changes represented in our selected material are consistent with this spectrum, although lamellar clubbing, which was represented in our samples, was not assessed in the previous studies. The clinical relevance of gill alterations is highly context-dependent and is influenced by their extent and anatomical location, particularly whether lamellar thickening increases the diffusion distance between blood and water and thereby impairs gas exchange [20].

In this study, we assessed the expression profiles of selected immunological (Cox-2b, IFN-α, IFN-γ, IL-1β, IL-8, SAA-5, IL-10, TGF-β, IL-4/13α, IgM, and IgT) and physiological (NKA α-1a, NKA α-1b, Rhcg1) markers, chosen for their established roles in immune regulation and gill cellular function. The results suggest a potential association between the expression of several immunological genes and histopathological features, such as lamellar clubbing, mucous cell proliferation, and lamellar inflammation, with elevated gene expression levels corresponding to higher histological scores.

Lamellar clubbing is an unspecific cellular thickening of the tip of lamellae consisting of proliferation and/or inflammation processes [43]. It is one of the first gill reactions to external irritants [44]. Lamellar clubbing is confined to the periphery of the lamellae and will thus have less impact on respiration. The outer marginal channel (OMC) travels the circumference of the lamellae and possesses the largest share of the lamellar surface area in contact with water [45]. Increased cellularity in this area could influence the lamellar blood transport balance and increase the load on/exposure of the lamellar sinusoids in the long run.

IFN-α is constitutively expressed in the gills, regulates inflammation, and exerts effects against viral and bacterial threats [46,47]. However, salmon kept in open cages in Lochs expressed higher levels than fish kept in RAS [48]. In salmonids, IFN-α functions as an immunostimulatory cytokine, orchestrating both innate and adaptive immune defenses, particularly against viral pathogens. While not pro-inflammatory in the classical sense, it is critical for effective immunity because it can amplify and direct immune responses. As inflammation is often found in the cellular thickening that constitutes lamellar clubbing, a connection between higher expression and clubbing cannot be ruled out. In this context, it was shown that salmon kept in RAS mounted a stronger inflammatory response to poly I:C (synthetic dsRNA) than fish kept in open cages [48], which was taken as an indication of less priming in pristine environments and was viewed as a compensatory response when exposed to pathogens. In line with this, higher IgT expression correlated with increased lamellar clubbing. IgT is produced by mucosal B cells and is essential for mucosal immunity, including gills [49,50]. Increased B cells, IgT expression, and IgT were demonstrated after bacterial infection [51]. Likewise, the observed association between IgT and lamellar inflammation may be linked to an increased inflamed area and, thus, a higher number of immune cells.

Mucous cells are typically found in low numbers in the gill lamellae of healthy fish, but their proliferation in response to gill irritation and disease is well documented [20,43,52]. Fewer than one mucous cell per lamella has been reported in healthy salmon during the freshwater phase [53]. In the selected freshwater material in the present study, only three filaments had more than one mucous cell per lamella. This observation is descriptive of the selected samples and should not be interpreted as a population estimate.

IL-4/13α is constitutively and highly expressed in salmonid gills, associated with Th2 immune responses, and involved in reactions to parasites [54]. A role for IL-4/13α in gill mucus production has been suggested [55], which aligns with the increased expression observed alongside mucous cell numbers in our study. The correlation between mucous cell numbers and NKA α-1a might be explained by the time elapsed since smoltification. This coincides with increased fish weights, which have previously been found to correlate with mucous cell numbers [53]. Na+/K + ATPase (NKA) exists in multiple isoforms on chloride cells in fish gills, with α-1a and α-1b isoforms having distinct roles under different environmental conditions. The expression of NKA α-1a, which is the dominant form in freshwater (ion uptake role), is expected to diminish in saltwater, where NKA α-1b is the dominant form (ion secretion role) [[56], [57], [58]]. α-1b levels can be detected in freshwater, whereas α-1a levels have been reported to be almost undetectable in Atlantic salmon a month after saltwater transfer [56,59]. In our study, α-1a was more highly expressed in freshwater than in saltwater, and α-1b showed higher expression than α-1a in saltwater. Still, surprisingly, both isoforms were detected in both environments and deviated from the expected pattern, with α-1b showing higher expression than α-1a in freshwater. Further, the isoform switching was not evident, and there are no apparent reasons for these findings.

Although the associations involving Cox-2b and IL-4/13α did not remain significant after FDR correction, their nominal patterns may be considered hypothesis-generating. Expression of Cox-2b and IL-4/13α was negatively and positively correlated with mucous cell numbers, respectively. This may suggest an inhibitory effect of IL-4/13α on Cox-2b expression, consistent with findings in mammals, where IL-4/13 has been reported to suppress Cox-2b activity [60]. In Atlantic salmon, Cox-2b is constitutively expressed in the gills, indicating an immunological state of preparedness against potential pathogens [28,61]. Cyclooxygenase enzymes, including Cox-2b, catalyze the conversion of arachidonic acid, liberated from cell membranes, into prostaglandins (PGs), which mediate inflammatory processes such as vasodilation and increased tissue perfusion in mammals [62]. The regulatory mechanisms linking Cox-2b and IL-4/13α expression to mucous cell dynamics remain to be elucidated. Nevertheless, the association of IL-4/13α with increased mucous cell numbers is notable, given that IL-4/13α is a hallmark of Th2-type immune responses and tissue preservation.

All fish tested negative for Ichthyobodo spp., *Paranucleospora theridion*, and *Saprolegnia* spp. Ca. *Branchiomonas cysticola* was the only agent detected and occurred at low to moderate levels, based on the semiquantitative scale described by Mitchell et al. [32], in fish from both production systems. Detection was limited to one fish group in each system and occurred in fewer than half of the selected FG2 samples and in approximately 100% of the selected FG4 samples. These sample-level detection frequencies should not be interpreted as facility-level prevalence estimates. Although no clinical signs of gill disease were observed, higher Ca. *Branchiomonas cysticola* loads coincided with increased lamellar inflammation scores. The selected, cross-sectional design cannot establish whether the bacterium contributed to the inflammation. Ca. *Branchiomonas cysticola* is commonly detected in freshwater- and saltwater-reared salmon [32,63], including fish without overt gill pathology [32]. Although increased loads have been linked to proliferative gill inflammation [32], more recent work has not demonstrated a consistent relationship between bacterial load and epithelial hyperplasia [42]. Detection of the bacterium in fish with no or only mild gill alterations is therefore consistent with previous observations.

A recent study investigated the relationship between gene expression and the presence of *Ca. Branchiomonas cysticola* and gill histopathology in presumptively healthy Atlantic salmon undergoing thermal delousing. However, no consistent patterns were observed [19], and mucous cells and lamellar clubbing were not assessed as separate parameters. The authors suggested that gene selection and sampling timing may have contributed to the absence of detectable correlations. Furthermore, the lack of association between gene expression and cellular changes was attributed to the well-established limitation that mRNA levels only partially reflect protein abundance due to post-transcriptional regulation [64]. While gene expression remains a widely used proxy for protein-level activity [64], and our study incorporated mucous cells and lamellar clubbing in the histological assessment, the limited correlations observed in our data likely reflect similar constraints. These findings underscore the need for complementary proteomic analyses in future studies to more accurately link transcriptional profiles to functional tissue responses.

Several limitations should be noted. First, fish from the freshwater and saltwater production systems originated from different facilities and differed in fish size, life stage, and sampling time. The water environment is therefore confounded with these factors, and differences between the systems cannot be attributed to water type alone. Second, we selected the final material partly based on histological suitability and the apparent presence or absence of gill changes to span a range of morphologies. The sample is consequently not representative of the source populations, and the observed frequencies, severity distributions, and between-system differences should not be interpreted as prevalence estimates or population reference values. Because selection was partly conditioned on the histological outcome, it may also influence the magnitude and uncertainty of the estimated gene-morphology associations. These associations should therefore be interpreted as exploratory relationships within the selected material rather than unbiased population effect estimates. Finally, given the number of markers and comparisons relative to sample size, only associations that remained significant after multiple-testing correction (Supplementary Table S1) should be regarded as comparatively robust and should still be validated in independent, prospectively sampled material.

## 5 Conclusion

This study should be interpreted as exploratory and characterizes mild gill alterations, selected transcript markers, and pathogen detection in a deliberately selected sample of apparently healthy Atlantic salmon from two commercial RAS production systems. Mild morphological changes were represented in fish without overt gill disease, but the sampling design does not establish how common these changes are in the source populations or define a normal population baseline. Within the selected material, mucous cell, lamellar clubbing, and lamellar inflammation scores were higher among fish from the saltwater production system; however, these descriptive differences cannot be attributed to water type because facility, fish size, life stage, and sampling time were confounded. The most robust associations after multiple-testing correction involved IFN-α and IgT with lamellar clubbing or inflammation, and IgM and NKA α-1a with mucous cell numbers. Ca. *Branchiomonas cysticola* was detected at subclinical levels and coincided with increased lamellar inflammation, but causality was not established. The findings illustrate the complexity of interpreting gill health from histopathology or transcript data alone and support the combined use of morphological, molecular, and pathogen data. Representative prospective studies are required to estimate the prevalence of mild gill changes and to validate the identified gene-morphology relationships.

## Declaration of competing interest

The authors declare the following financial interests/personal relationships which may be considered as potential competing interests: Thomas Amlie is employed by DNV, which provides clinical and diagnostic services to the fish farming industry.

## Figures and Tables

**figs1:**
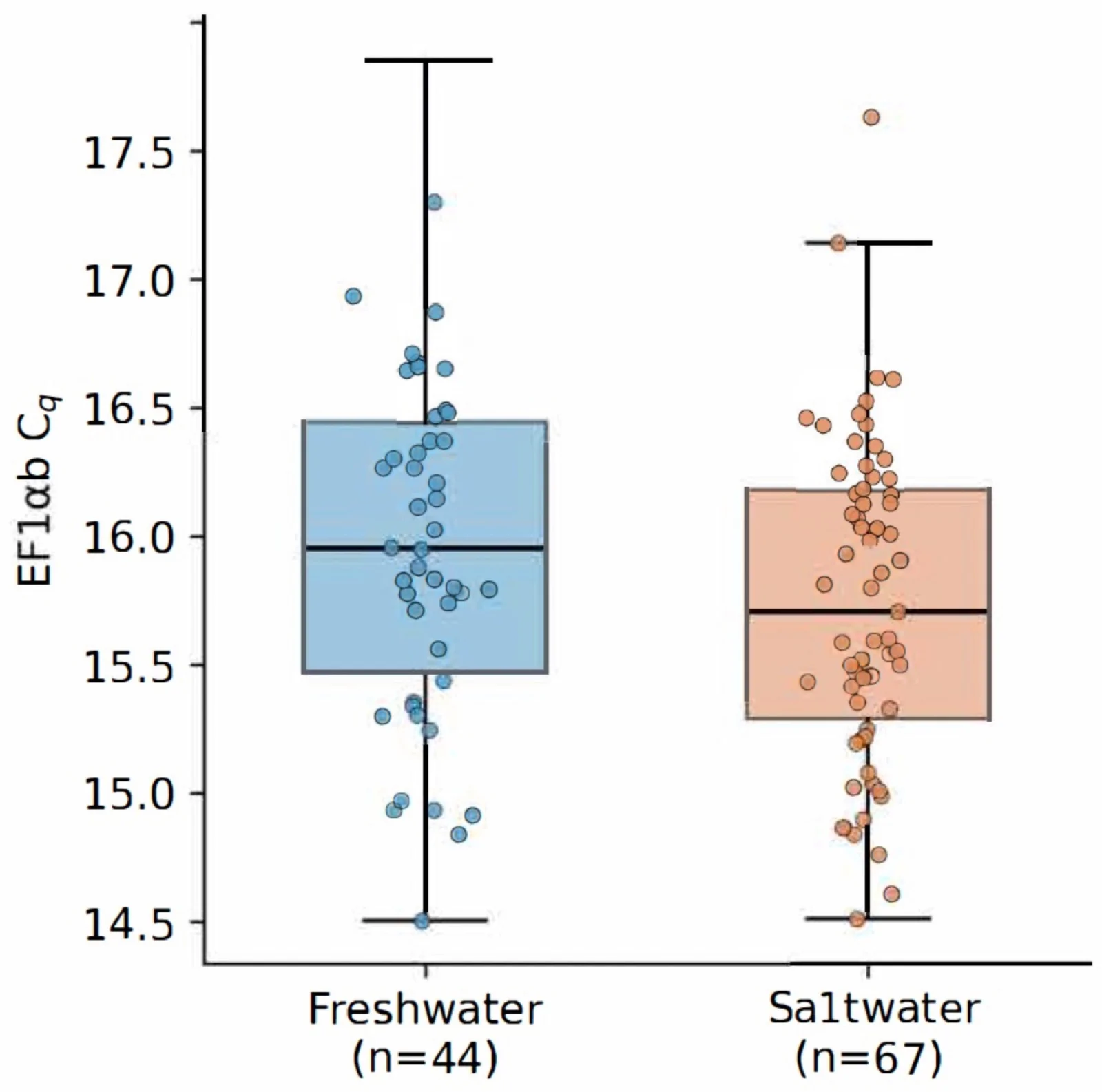


**figs2:**
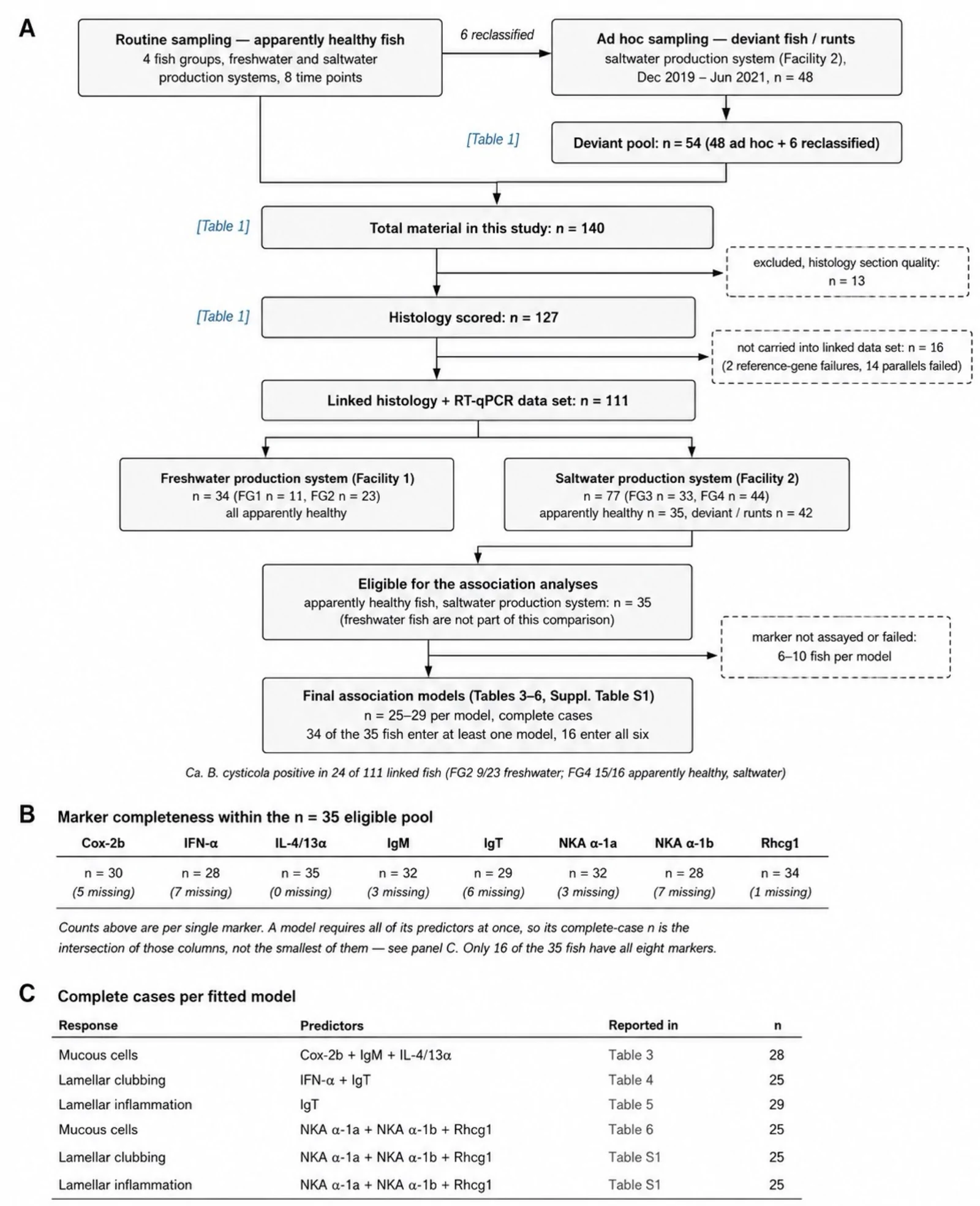


## Data Availability

Data will be made available on request.
