# Supplementary material for: Concurrent analysis of histomorphology and immune-related gene expression in gills of Atlantic salmon (Salmo salar) from recirculating aquaculture systems
Source: Biochem Biophys Rep. 2026 Oct 3;48:102808. doi: 10.1016/j.bbrep.2026.102808 (PMC13661231; doi:10.1016/j.bbrep.2026.102808)
Supplement: Supplementary file 1 [file mmc1.docx]

| Response | Model | Predictor | N | Coefficient | p value (raw) | q value post-hoc (Benjamini–Hochberg) | Survives FDR* |
| --- | --- | --- | --- | --- | --- | --- | --- |
| club_sum | neg. binomial | IFNa | 25 | -0.879 | 0.000 | 0.000 | yes |
| laminf_sum | neg. binomial | IgT | 29 | -0.290 | 0.000 | 0.003 | yes |
| muc_sum | neg. binomial | IgM | 28 | -0.203 | 0.001 | 0.004 | yes |
| muc_sum | linear | NKAa1a | 25 | -1.909 | 0.002 | 0.007 | yes |
| club_sum | neg. binomial | IgT | 25 | -0.248 | 0.010 | 0.030 | yes |
| laminf_sum | linear | NKAa1a | 25 | -0.665 | 0.034 | 0.076 | no |
| muc_sum | neg. binomial | IL413a | 28 | -0.320 | 0.036 | 0.076 | no |
| muc_sum | neg. binomial | Cox2b | 28 | 0.220 | 0.050 | 0.095 | no |
| laminf_sum | linear | Rhcg1 | 25 | 0.624 | 0.336 | 0.553 | no |
| club_sum | linear | NKAa1b | 25 | -1.302 | 0.384 | 0.553 | no |
| muc_sum | linear | Rhcg1 | 25 | 0.932 | 0.432 | 0.553 | no |
| laminf_sum | linear | NKAa1b | 25 | -0.336 | 0.442 | 0.553 | no |
| club_sum | linear | Rhcg1 | 25 | -1.235 | 0.574 | 0.662 | no |
| muc_sum | linear | NKAa1b | 25 | 0.186 | 0.815 | 0.874 | no |
| club_sum | linear | NKAa1a | 25 | 0.062 | 0.951 | 0.951 | no |
